# Draft Genome Sequence of *Streptacidiphilus oryzae* TH49^T^, an Acidophilic Actinobacterium Isolated from Soil

**DOI:** 10.1128/genomeA.00703-15

**Published:** 2015-06-25

**Authors:** Yu Ri Kim, Sewook Park, Tae-Su Kim, Min-Kyeong Kim, Ji-Hye Han, Yochan Joung, Seung Bum Kim

**Affiliations:** Department of Microbiology and Molecular Biology, Chungnam National University, Daejeon, Republic of Korea

## Abstract

The draft genome sequence of *Streptacidiphilus oryzae* strain TH49^T^, an acidophilic actinobacterium, was obtained. The draft is composed of six scaffolds totaling 7.8 Mbp, and it contains 6,829 protein-coding genes and 91 RNA genes. Genes related to respiratory nitrate reduction, siderophore production, and biosynthesis of other secondary metabolites were identified.

## GENOME ANNOUNCEMENT

The family *Streptomycetaceae* was proposed by Waksman and Henrici (1) and comprises 3 genera, *Streptomyces*, *Kitasatospora*, and *Streptacidiphilus* (1, 2). The genus *Streptacidiphilus* is the most recently described member among them and currently encompasses 10 acidophilic species, which typically grow in a pH range between 3.5 and 6.5, with optimum values around pH 4.5 to ~5.5 (2). *Streptacidiphilus* is widely distributed in a variety of terrestrial habitats, such as coal mine waste, acid mine drainage soils, and acidic forest soils (3–5). Acidophilic actinobacteria, such as streptacidiphili, are considered a good source of antibiotics and acid-stable enzymes (6, 7). Soil acidophilic actinobacteria may have higher potential than their neutrophilic counterparts for some activities, for example, antifungal activity (8, 9).

Recently, the genome sequences of selected streptacidiphili, *S. albus* NBRC 100918^T^, *S. anmyonensis* NBRC 103185^T^, *S. carbonis* NBRC 100919^T^, *S. jiangxiensis* NBRC 100920^T^, *S. melanogenes* NBRC 103184^T^, and *S. neutrinimicus* NBRC 100921^T^, were analyzed. In this study, the genome sequence of *Streptacidiphilus oryzae* was determined, and some basic features were analyzed.

*S. oryzae* TH49^T^ is an isolate from an acidic rice field soil sample and grows optimally at pH 5.0 to ~5.5 (10). The biomass of *S. oryzae* TH49^T^ (NCBI taxonomy identification no. 1449353) was obtained from a culture in ISP2 broth after incubation at 30°C for 3 days. High-quality genomic DNA was prepared using a DNA prep kit (SolGent), and the construction of a DNA library by PacBio 10-kb and sequencing on a PacBio RS platform (Pacific Biosciences) were performed at the Joint Genome Institute (JGI).

The analyzed draft genome sequence is composed of 6 DNA scaffolds covering 7,806,543 bp, with 73.36% G+C content, and contains 6,829 protein-coding genes and 91 RNA genes. The number of copies of rRNA genes for each subunit was between 7 and 8. *S. oryzae* TH49^T^ possessed genes related to anaerobic respiration, namely, the alpha, beta, and gamma subunits of respiratory nitrate reductase and respiratory nitrate reductase chaperone.

Genes for the biosynthesis of secondary metabolites were found in the genome, including genes for siderophore production and those related to the synthesis of type II polyketides, terpenoid backbones, and aminoglycosides.

### Nucleotide sequence accession number.

The annotated draft genome sequence of *S. oryzae* TH49^T^ has been deposited in DDBJ/EMBL/GenBank under the accession no. JQMQ00000000.
